# Exposure of Individuals in Europe to Air Pollution and Related Health Effects

**DOI:** 10.3389/fpubh.2022.871144

**Published:** 2022-05-25

**Authors:** Naixin Li, Rainer Friedrich, Christian Schieberle

**Affiliations:** Department Technology Assessment and Environment, Institute of Energy Economics and Rational Energy Use, University of Stuttgart, Stuttgart, Germany

**Keywords:** PM2.5, NO_2_, exposure to air pollution, socio-demographic status, health impacts related to air pollution

## Abstract

Air pollutants, especially PM2.5 and NO_2_, are associated with adverse health impacts, as shown by numerous epidemiological studies. In these studies, the observed health impacts have been correlated with ambient concentrations, mainly taken from air pollution monitoring stations. However, individuals are harmed by the pollutants in the inhaled air at the places where they stay, and thus, the concentration of pollutants in the inhaled air is obviously a better indicator for health impacts than the ambient concentration at a monitoring station. Furthermore, the current method for estimating the occurrence of chronic diseases uses annual average concentrations as indicator. However, according to current hypotheses, chronic diseases, especially chronic mortality, develop through the exposure to pollutants over many years, maybe up to a full lifetime. Thus in this study, a methodology and a computer-aided probabilistic model system are described for calculating the exposure of a person to PM2.5 and NO_2_ over the whole lifetime where the person is characterized by attributes such as age, gender, place of residence and work as well as socioeconomic status. The model system contains a “life course trajectory model”, which estimates the course of the education and professional development for the past lifetime of a person, whose present socioeconomic status is known. Furthermore, a “time-activity model” estimates at which places (so-called micro-environments) a person with a certain socioeconomic status stayed and how long he stayed there within a certain year. The concentrations of air pollutants in indoor environments are calculated with a “mass-balance model”, the outdoor concentrations with “atmospheric models”. Finally, the results of these models are combined to estimate the annual average exposure for the life years of individuals and population subgroups. The exposure is then used to estimate and monetize health impacts. The exposures and health impacts for a number of population subgroups in Europe are presented. For instance, a European citizen, who was 70 years old in 2015, has been exposed to around 25 μg/m^3^ of PM2.5 during his lifetime above the age of 30, which is associated with a reduction of life expectancy of 13.4 days per year of exposure above 30.

## 1. Introduction

The long term exposure to air pollutants, especially to fine particles (PM2.5) and nitrogen dioxide (NO_2_), is associated with a considerable reduction in the life expectancy of the exposed population. This has been proven by a growing number of epidemiological studies. One of the first studies was published by (1). In this study, the authors found a negative correlation between chronic mortality and long term exposure to fine particles. Meanwhile, a larger number of studies have been published, that confirm various negative health impacts associated with exposure to PM2.5 and NO_2_ (2–6).

Traditionally, concentration response functions (CRFs) are derived from epidemiological studies that characterize the health impacts caused by PM2.5 and NO_2_. These functions quantify how many additional illnesses (e.g., chronic bronchitis) occur, or how many years of life are lost due to premature deaths per year, if 100,000 people are exposed to 1 μg/m^3^ higher background concentrations for 1 year. However, the drawbacks of this methodology should be noticed.

First of all, the CRFs correlate the health impacts with ambient pollutant concentrations, which in most cases were measured by nearby air monitoring stations. However, the measurement data at monitoring stations are not able to reflect the real exposure a person suffers since people stay in different micro-environments—and not near a monitoring station—during the day. In fact, most people spend the majority of their time in indoor micro-environments such as home, workplace, school, etc. This means, the utilization of ambient pollutant concentrations ignores the influences of indoor pollutant sources, time activity pattern and building characteristics.

Furthermore, the CRFs for estimating the occurrence of chronic diseases use annual average concentrations as indicator. However, according to current hypotheses the health status of a person, especially regarding chronic diseases, is affected by exposure to pollutants over many years.

In addition, each CRF uses mass concentrations of one single pollutant as indicator. However, the impacts might be influenced by a combination of several pollutants (e.g., NO_2_ and PM2.5), by other factors than pollutant concentrations (e.g., socioeconomic parameters) and in the case of particles by the number, size and species contained.

Obviously, a prerequisite for developing new impact functions, that overcome these weaknesses would be to provide indicators, that express the exposure to all relevant pollutants over the whole lifetime of persons, whose socioeconomic status are known.

Thus, a probabilistic model system has been developed and described in this paper for estimating the exposure (i.e., the concentration in the inhaled air) to PM2.5 and NO_2_ over the whole lifetime for a person living in Europe, including EU27^1^ countries plus Norway and Switzerland (EU27+2). The exposures of people to both pollutants are affected by the attributes of the socioeconomic status (SES) (7–9). The developed methodology can thus be applied to persons that are characterized by features such as age, gender, place of residence and work, and SES factors like income level, employment status and education level.

The result of applying the exposure modeling system is a probability distribution of exposure to air pollutants of persons with certain features as described above over their full lifetime.

To demonstrate the application of the result for estimating associated health impacts, the existing CRFs are used.

The available CRFs have to be transformed into exposure response functions (ERFs), to be able to use the exposure as indicator for health effects. Finally, the health impact endpoints are on the one hand transferred into DALYs (disability adjusted life years) as an aggregated measure for the different health impacts. On the other hand, they are monetized using contingent valuation (e.g., willingness to pay studies) to have an aggregated value of the health impacts (“damage costs”), that can be directly compared with costs.

The exposure modeling methodology developed here is a prerequisite for being able to develop advanced methods for estimating health impacts, especially for developing improved ERFs.

## 2. Methods and Data

### 2.1. Overall Structure of the Methodology

During a certain period of time, e.g., a year, a person stays in different “locations” with, respectively, different pollutant concentrations. These “locations”, which are assumed to be a chunk of air space with homogeneous pollutant concentration, are defined as “micro-environments” (10). In this study, the micro-environment approach is applied to simulate the exposure of individuals, which is defined as Equation (1):


(1) $$\begin{array}{l} EXP=\frac{\overset{n}{\underset{i=1}{\sum }}C_{i}T_{i}}{365\times 24\text{h}} \end{array}$$

where *EXP* is the average personal exposure during the year under study [μg/m^3^], *C*_i_ is the concentration in micro-environment *i* [μg/m^3^], while the person is staying in the micro-environment and *T*_i_ is the time spent in the micro-environment *i* [h] during the year.

**Figure 1:**
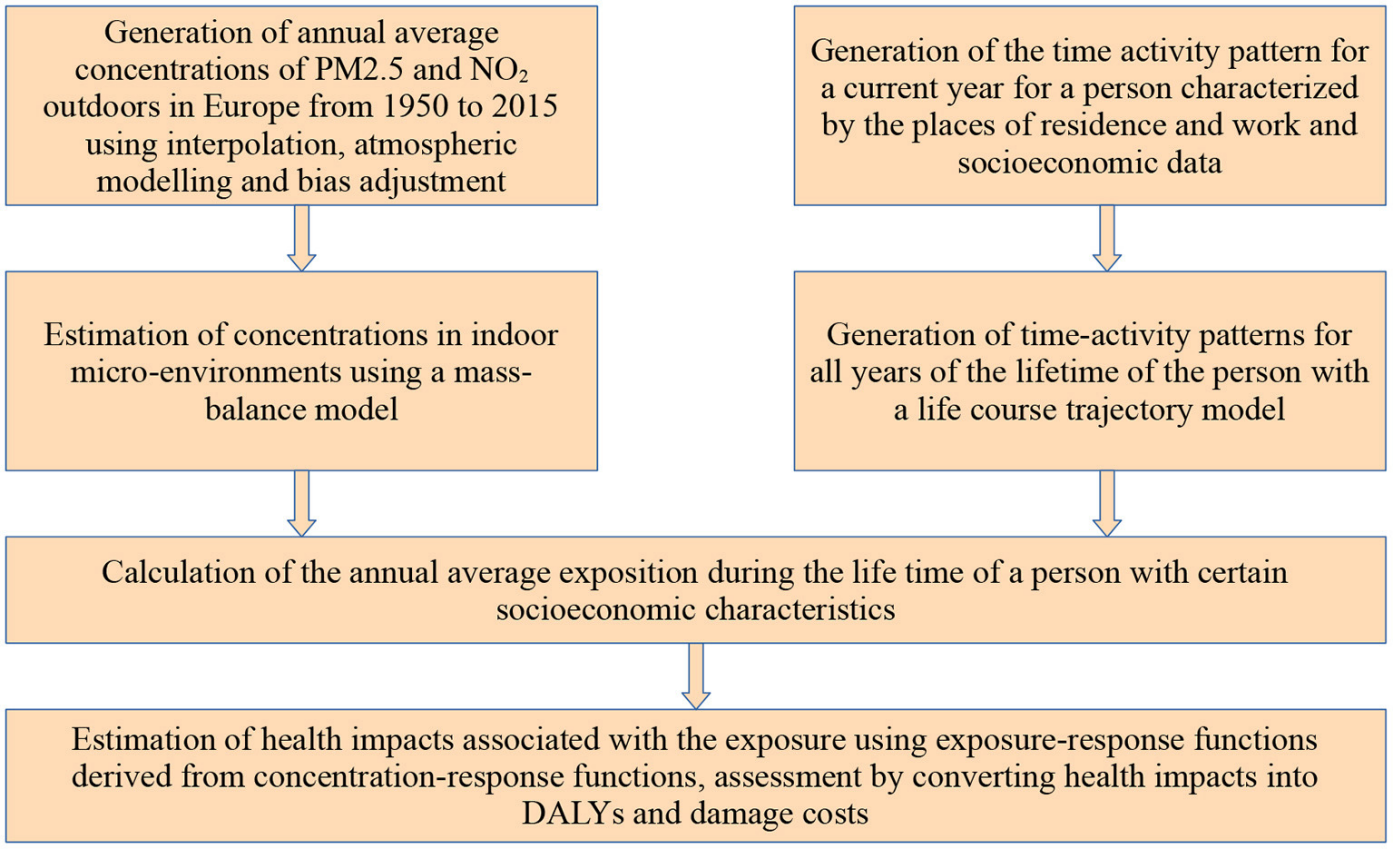
Overall structure of the methodological framework.

Within the frame of this study, a comprehensive probabilistic model system for assessing the temporal course of the external exposure for population subgroups that are characterized by certain features (e.g., age, gender, socioeconomic status) has been developed. The overall structure of the methodology applied in this study is displayed in Figure 1.

In the first step of applying the modeling system, annual averaged concentrations of the pollutants PM2.5 and NO_2_ in the ambient environment are estimated for the whole Europe and all years from 1950 to 2015 in a sufficient resolution to distinguish between rural and urban areas (Section 2.2.1); so the concentrations in outdoor micro-environments are provided. In a second step, using the outdoor concentration data and data on the indoor emissions and on ventilation, a mass-balance model is used to estimate the pollutant concentrations in different indoor micro-environments (Section 2.2.2), again for the years from 1950 to 2015. In the next step, the time activity pattern of individuals with certain socioeconomic features (e.g., place of residence, work or educational facility, gender, age, income level, occupational status) is generated (Section 2.3), hence the question is answered, in which micro-environment these individuals stayed in a year and how long they stay there. As the exposure for the whole life time is needed, a life course trajectory model is used to predict, starting from the time-activity matrix of a person in a current year determined in the previous step, time-activity matrices for all previous years of the person's lifetime (Section 2.4). The last step is to calculate the annual averaged exposure for the whole lifetime of a person with certain socioeconomic features. To do this, the concentration in each micro-environment where the person stays is weighted by the length of stay (Equation 1 in this Section). To give an example for applying the results of the exposure modeling, the calculated exposure data is used together with modified existing CRFs and population data to assess the health impacts of the European population related to exposure to PM2.5 and NO_2_ (Section 2.5).

The modeling system is stochastic. For all the parameters of each used model a probability distribution of the parameter value is estimated. Then the Monte-Carlo method is used, i.e., for each run of the modeling system a large number of simulations with different parameter values is made resulting in a probability distribution of the result (i.e., exposure and health impacts).

Furthermore, model uncertainties exist, as models are simplified representations of reality. Important simplifications are discussed in the description of the models in the following chapters.

### 2.2. Concentration in Micro-Environments

The micro-environments taken into account in this study are home indoor, work indoor, school indoor, outdoor, travel/commute and other indoor environments. As displayed by Equation (1), two parts of information are necessary for assessing the exposure of individuals: the pollutant concentration in each micro-environment and the time spent in each micro-environment.

#### 2.2.1. Outdoor

The pollutant concentrations in as well indoor as outdoor micro-environments are highly influenced by the outdoor air concentration (see Equation 3, Equation 4 and Equation 11). Thus the first step of the methodology is to generate the ambient concentration fields for all European countries and all past years since 1945. In principle, monitoring data are used and interpolated, and results of calculations with atmospheric models are bias-adjusted. Several models and methodologies have been used, including the EMEP chemistry transport model (11), the EIONET interpolation method (12), the multiplicative bias adjustment (13), the EDGAR-HYDE emission data (14) and the EcoSense model (15–19).

##### 2.2.1.1. EMEP/MSC-W Chemical Transport Model

The chemical transport model (CTM) is used to simulate the three-dimensional mechanism of chemical distribution in the air. For European countries, EMEP/MSC-W is one of the most powerful CTMs for simulating the concentration fields for air pollutants including PM2.5 and NO_2_.

The concentration fields from 1980 to 2015 for European countries were directly available from Norwegian Meteorological Institute^2^. However, Bessagnet et al. (20) and Schaap et al. (21) have evaluated the performance of the EMEP model and reported a systematic underestimation of PM2.5 concentrations. To adjust the modeling results from EMEP, two methods have been adopted, the EIONET interpolation method and the multiplicative bias adjustment.

##### 2.2.1.2. EIONET Interpolation

Horálek et al. (22) developed an interpolation methodology to modify the EMEP concentration fields. This method incorporated a linear regression model between monitoring data from AirBase^3^ and additional data and the interpolation of residuals of the regression model. The additional data include EMEP/MSC-W model results, meteorological data, altitude and population density. For NO_2_ concentration fields, the CORINE Land Cover (CLC) data (23) and the OpenStreetMap^4^ data were additionally used (24). This method has improved the quality of the concentration fields from the EMEP model effectively. However, this method is rather constrained due to the availability of the monitoring data. Thus, this method was only applied to adapt the EMEP data for PM2.5 after 2005 and for NO_2_ after 2000.

##### 2.2.1.3. Multiplicative Bias Adjustment

The multiplicative bias correction has been widely used to modify the simulation results of CTMs (13). This method is described with Equation (2):


(2) $$\begin{array}{l} C_{\text{corrected}}=R_{\text{bias}}\times C_{\text{model}} \end{array}$$

where *C*_corrected_ is the corrected concentration [μg/m^3^], *R*_bias_ is the multiplicative bias adjustment factor, *C*_model_ is the originally modeled concentration [μg/m^3^].

In this study, this method has been applied to adapt the concentration fields from EMEP between 1980 and 2000s, as for these years no interpolated maps were available. The multiplicative bias adjustment factors were developed by the authors based on the EIONET concentration fields generated in Section 2.2.1.2.

Using bias correction factors, that have been calculated for years after 2000, for years before 2000 adds some additional uncertainties, especially as emissions change caused by the upheaval in Eastern Europe and a growing reduction of emissions from large emitters. However, we assume that most of these changes are mapped in the CTM model, so that using the bias factors still improves the result.

##### 2.2.1.4. EDGAR-HYDE and EcoSense

Concentration fields before 1980 were generated with the emission data from EDGAR-HYDE (Emission Database for Global Atmospheric Research—Hundred Year Database for Integrated Environmental Assessment) (7). EDGAR-HYDE provides anthropogenic emissions of CO_2_, CH_4_, N_2_O, CO, NO_x_, NMVOC, SO_2_, and NH_3_ from 1890 to 1990 with a temporal resolution of 10 years. The EcoSense model uses the source receptor matrices from EMEP/MSC-W to simulate the background concentrations fields (15–19), which were further multiplicative bias adjusted with the factors generated in Section 2.2.1.3.

#### 2.2.2. Indoor

##### 2.2.2.1. Indoor Concentration Modeling

For the indoor micro-environments, an average pollutant concentration is calculated with a mass-balance model (25). The model can be expressed as Equation (3):


(3) $$\begin{array}{l} C_{\text{in}}=\frac{C_{\text{out}}p\times AER+\frac{\overset{n}{\underset{i=1}{\sum }}E_{i}}{V}}{AER+k} \end{array}$$

where *C*_in_ is the indoor concentration [μg/m^3^], *C*_out_ is the ambient pollutant concentration [μg/m^3^], *p* is the penetration factor, *AER* is the air exchange rate [h^-1^], *k* is the decay rate [h^-1^], *E*_i_ is the emission rate of source *i* [μg/h], and *V* is the apartment volume [m^3^].

For determining the size and volume of the apartments the EU-SILC database is used (26). The data are summarized and stratified by country and other SES variables, including income level, degree of urbanization (urban or rural areas) and civil status. It is shown by the data that the SES variables are important affecting factors of the room size (27).

It should be noticed that most of the existing mass-balance models simulate the concentration generated from cooking based on the assumption that the emission spread out evenly over the entire living space. However, according to Huboyo et al. (28) and Poon et al. (29), the concentration in the kitchens were found to be higher than in other rooms of the same residence. The reason behind is that many kitchen are “isolated” from other rooms of the living space during cooking since people keep the door of the kitchen closed and partly use cooker bonnets. However, in the case of open plan kitchens, aerosols emitted from cooking can diffuse rather rapidly to the adjacent living space. Considering the information stated above, the living area affected by cooking has been assumed as residence area multiplied with a random factor ranging from 0.2 to 0.9.

For the indoor micro-environment, ventilation is an important in determining the air quality. We distinguish between natural and mechanical ventilation systems. The natural ventilation system is very common in old buildings. For such buildings, the air exchange is mainly realized by infiltration and exfiltration through opening windows or doors, but also through small leaks in closed windows. Since the last few decades, many of those old buildings have been insulated and air-tight windows and doors have been installed to avoid unnecessary energy losses. New tight windows save energy, but also lead to a reduction of the air exchange rate and thus result in poorer indoor air quality (30). Many studies have addressed the insufficient air exchange rate by retrofitted buildings (31, 32).

The installation of mechanical ventilation systems is a solution for improving the air quality of insulated buildings. Such systems are equipped with powered air movement devices to accelerate the air exchange with the ambient environment. A mechanical ventilation system is usually accompanied with heat recovery and sometimes also with particle filters to purify the air.

With an advanced version of mechanical ventilation, a supply and a return air fan are installed to recirculate parts of the air. This system is usually accompanied with an air handling unit (AHU), which contains fans, filters, heating and cooling elements, and other equipment for air circulation, heat recovery and humidifying or dehumidifying. Compared to other ventilation systems, AHU can remove the pollutants from indoor sources efficiently due to its adequate air exchange rate. Moreover, the circulation of air through the filters in the AHU also stimulates the removal of air pollutants.

In order to take the influence of the ventilation system on the indoor air pollutant concentration into account, the buildings are classified into four categories: without insulation and mechanical ventilation (“original”), thermally “insulated” with tight windows, mechanically ventilated with heat recovery (“mechanical”) and “AHU”. Considering the effect of recirculation, Equation (3) for buildings equipped with AHU is modified by (33) as:


(4) $$\begin{array}{l} C_{\text{in}}=\frac{C_{\text{out}}p\times AER+\frac{\overset{n}{\underset{i=1}{\sum }}E_{i}}{V}}{AER+k+\eta ND} \end{array}$$

where η is the removal efficiency of filter, *N* is the recirculated air exchange rate [h^-1^] (measurement of how much air is removed from a space and reused in a given time period) and *D* is the duty cycle of AHU (fraction of time that the AHU fan is in operation).

Equations (3) and (4) can be transformed into the following Equation (5):


(5) $$\begin{array}{l} C_{\text{in}}=F_{\text{inf}}C_{\text{out}}+C_{\text{ig}} \end{array}$$

where *F*_inf_ is the infiltration factor and *C*_ig_ is the concentration generated from indoor sources [μg/m^3^]. As interpreted by Equation (5), the indoor air pollutant concentration comprises two components, the infiltration of pollutants with the outdoor air and the air pollutants generated from indoor sources.

##### 2.2.2.2. Indoor Sources

In this study, environmental tobacco smoke (ETS), cooking, wood burning, candles/incense sticks and other activities were identified as the most important indoor sources.

Smoking has been considered as one of the most important sources by numerous studies (34, 35). The emissions from cigarettes are calculated with Equation (6):


(6) $$\begin{array}{l} E_{\text{cig},i}=\frac{S_{\text{cig}}N_{\text{cig},i}}{T_{i}} \end{array}$$

where *E*_cig_,_i_ is the emission rate for cigarettes in micro-environment *i* [μg/h], *S*_cig_ is the source strength per cigarette [μg], *N*_cig_,_i_ is the number of cigarettes smoked in the micro-environment *i* per day and *T*_i_ is the total time in micro-environment *i* during a day [h].

The percentage of smokers and the number of cigarettes consumed per day dependent on country, gender, age, income level, employment status and further parameters is derived from the European Health Interview Survey (EHIS) (36). The health impacts due to smoking have caught the attention in Europe since a few decades and a series of measures for smoking control have been implemented. In this study, it is assumed that people were less exposed to second-hand smoking after the 2010s thanks to the smoking bans implemented in public areas in European countries.

Cooking has been proven as another crucial indoor source for PM2.5 and NO_2_ (37, 38). The pollutants generated from cooking can however be mitigated by a kitchen hood. The emissions from cooking are calculated with Equation (7):


(7) $$\begin{array}{l} E_{\text{cooking},i}=\frac{S_{\text{cooking}}t_{\text{cooking},i}\left(1-CE\right)}{T_{i}} \end{array}$$

where *E*_cooking, i_ is the emission rate for cooking in micro-environment *i* [μg/h], *S*_cooking_ is the source strength of cooking [μg/min], *t*_cooking, i_ is time of cooking activities in micro-environment *i* per day [min], *CE* is the capture efficiency of kitchen hood and *T*_i_ is the total time in micro-environment *i* during a day [h].

The time spent in the kitchen is taken from the time-activity patterns (see Section 2.3 and 2.4), in which it is reported as a function of age, gender, employment status, family status, education, and income level.

Wood combustion is a source of a large amount of indoor pollutants including particles and NO_2_ (39, 40). Hartinger et al. (41) have reported the extraordinary mitigation effect of chimneys, which can lead to over 95% of the pollutants generated from fireplaces to outdoor environment. The emission due to wood burning is calculated with Equation (8):


(8) $$\begin{array}{l} E_{\text{wood},i}=\frac{S_{\text{wood}}t_{\text{wood},i}H_{\text{demand}}V_{i}\left(1-R_{\text{removal}}\right)}{T_{i}} \end{array}$$

where *E*_wood, i_ is the emission rate for wood burning in micro-environment *i* [μg/h], *S*_wood_ is the source strength of wood burning [μg/kJ], *t*_wood, i_ is the time of burning wood in micro-environment *i* per day [h], *H*_demand_ is the heat demand [kJ/(m^3^h)],*V*_i_ is the room volume of micro-environment *i* [m^3^], *R*_removal_ is the removal ratio of chimney and *T*_i_ is the total time in micro-environment *i* during a day [h].

The proportion of apartments with wood-burning stoves or open chimneys in the living room per country is taken from (42). From about 2010 on, newer stoves emit considerably less pollutants into the interior (43).

The emissions from candles/incense are calculated with Equation (9):


(9) $$\begin{array}{l} E_{\text{candle},i}=\frac{S_{\text{candle}}t_{\text{candle},i}}{T_{i}} \end{array}$$

where *E*_candle, i_ is the emission rate for candles and incense sticks in micro-environment *i* [μg/h], *S*_candle_ is the source strength of the candle/incense [μg/min], *t*_candle, i_ is the time for burning candles in micro-environment *i* per day [min] and *T*_i_ is total time spent in micro-environment *i* during a day [h]. Other activities than those mentioned above are captured by Equation (10):


(10) $$\begin{array}{l} E_{\text{other},i}=\frac{\overset{n}{\underset{j=1}{\sum }}S_{\text{other},j}t_{\text{other},i,j}}{T_{i}} \end{array}$$

where *E*_other, i_ is the emission rate for other activities in micro-environment *i* [μg/h], *S*_other, j_ is the source strength of activity *j* [μg/min], *t*_other, i, j_ is the time spending on activity *j* in micro-environment *i* per day [min] and *T*_i_ is the total time in micro-environment *i* during a day [h].

Tables 1, 2 list the data employed in this study for the mass-balance model. The air exchange rates of original buildings were taken from (44); different values for Southern, Eastern, Northwestern and Northern Europe are applied. Please note that the data in Table 1 is averaged for the whole year, where in summer the windows are open more often than in winter.

**Table 1:** Values of air exchange rate for micro-environment “work” categorized as “original”, “insulated”, “mechanical”, and “AHU” buildings.

**Parameter**	**Micro-environment**	**Pollutant**	**Building type**			
			**“Original”**	**“Insulated”**	**“Mechanical”**	**“AHU”**
*a* [h^-1^]	Home	-	Northwestern: 0.83 (± 0.46), log-normal; Southern: 1.29 (± 1.09), log-normal; Eastern: 0.75 (± 0.43), log-normal; Northern: 0.81 (± 0.85), log-normal	0.35 (± 0.15), log-normal	0.50 (± 0.30), log-normal	
	Work	-	(0.1, 0,6, 1.8), triangular	(0.1, 0.3, 0.8), triangular	(0.5, 1.4, 5.0), triangular	0.5 (± 0.3), log-normal
*p*	-	PM2.5	0.95 (± 0.30), log-normal	0.95 (± 0.30), log-normal	0.75 (± 0.20), log-normal	0.75 (± 0.20), log-normal
		NO_2_	1, consistent	1, consistent	0.70 (± 0.12), log-normal	0.70 (± 0.12), log-normal
*k*	-	PM2.5	0.39 (± 0.10), log-normal	0.25 (± 0.10), log-normal	0.30 (± 0.15), log-normal	0.30 (± 0.15), log-normal
		NO_2_	0.87 (± 0.30), log-normal	0.65 (± 0.15), log-normal	0.75 (± 0.25), log-normal	0.75 (± 0.25), log-normal
η	-	PM2.5	0.10–0.70, uniform			
		NO_2_	0.25–0.90, uniform			
*N* [h^-1^]	-	-	5 (± 2), log-normal, *N* ≤ 25			
*D*	Home	-	Residential: 0-1.0, uniform			
	Other		0.5, constant			

**Table 2:** Values of source strength.

**Activity**	**Pollutant**	**Range of values and shape of the distribution function**
Cooking [μg/min of cooking]	PM2.5	1125 (± 280), normal
	NO_2_	Electric: 270 (± 75), normal; Gas: 1800 (± 450), normal
Wood burning [μg/kJ of wood burnt]	PM2.5	13–146, uniform
	NO_2_	58-185, uniform
Smoking per cigarette [μg]	PM2.5	10,950 (± 2,000), normal
	NO_2_	1,930 (± 65), normal
Candles/Incense [μg/min]	PM2.5	5.5–910, uniform
Set table, wash/put away dishes [μg/min]	PM2.5	20–180, uniform
Cleaning/other domestic work [μg/min]	PM2.5	90–440, uniform
Laundry, ironing, clothing repair [μg/min]	PM2.5	20–180, uniform
Imputed personal or household care [μg/min]	PM2.5	20–80, uniform
Wash, dress, care for self [μg/min]	PM2.5	20–80, uniform

#### 2.2.3. Travel

For the concentration of pollutant in the micro-environment travel, a traffic factor is applied as shown by Equation (11):


(11) $$\begin{array}{l} C_{\text{trans}}=F_{\text{ME}}\times C_{\text{out}} \end{array}$$

where *C*_trans_ is the concentration in transport [μg/m^3^], *C*_out_ is the ambient pollutant concentration [μg/m^3^] and *F*_ME_ is the factor used for the transport micro-environment (ME-factor).

This factor defines the ratio between the pollutant concentration in the means of transport and the background concentration. A large range for the micro-environment factor has been reported since the pollutant concentration in traffic is affected by multiple factors such as traffic modes, type of vehicles, type of roads and ventilation in vehicles. This traffic factor is summarized based on data from (45), (46), (47), and (48) and assumed to be 2 (± 1.7) and 2.5 (± 2.1) as normal distributed for PM2.5 and NO_2_, respectively. This rather simple approach was used as the contribution of transportation to the overall exposure is relatively limited. The time spent in the different transport modes is generated from the time-activity patterns (Sections 2.3 and 2.4), where they are reported as a function of age, gender, employment status, family status, education and income level. According to these data, the time spent on commuting per day is around 30 min, which is much less than the time spent at home, in offices, schools, etc., so that the uncertainties related to transport are less determinant for the result. However, in future developments of the model, differentiation by mode of transport could improve accuracy and allow the impact of transport model choice to be studied.

### 2.3. Time Activity Patterns

As shown in Equation (1), to simulate the exposure of a person to a certain pollutant, information about the places and the duration of stay of this person, which means time-activity data, is necessary. In this study, the time activity data were derived from the Multinational Time Use Study (MTUS) (49), in which multi-national harmonized sets of time use surveys were collected and analyzed. The dataset contains a large number of diary days from over 70 randomly sampled national-scale surveys with a standardized format. Each diary is a 24h time-activity profile that records the sequence of time, activity and micro-environment by a survey respondent. Additionally, some context variables, such as age, gender, employment status, family status, education, etc. were also recorded for each diary of the survey respondents. These variables have been used to assign the time activity diaries to the population subgroup (see Section 2.5.3) with the same features. As mentioned above, the model parameters for pollutant concentrations in micro-environments were given in the form of a certain probability distribution (see Section 2.2). For each diary, a large number of realizations were conducted for these parameters and a Monte Carlo analysis was carried out to assess the exposure to both air pollutants for individuals or population subgroups with certain SES characteristics. The exposure modeling then results in a probability distribution of the exposure (see Section 3).

**Figure 2:**
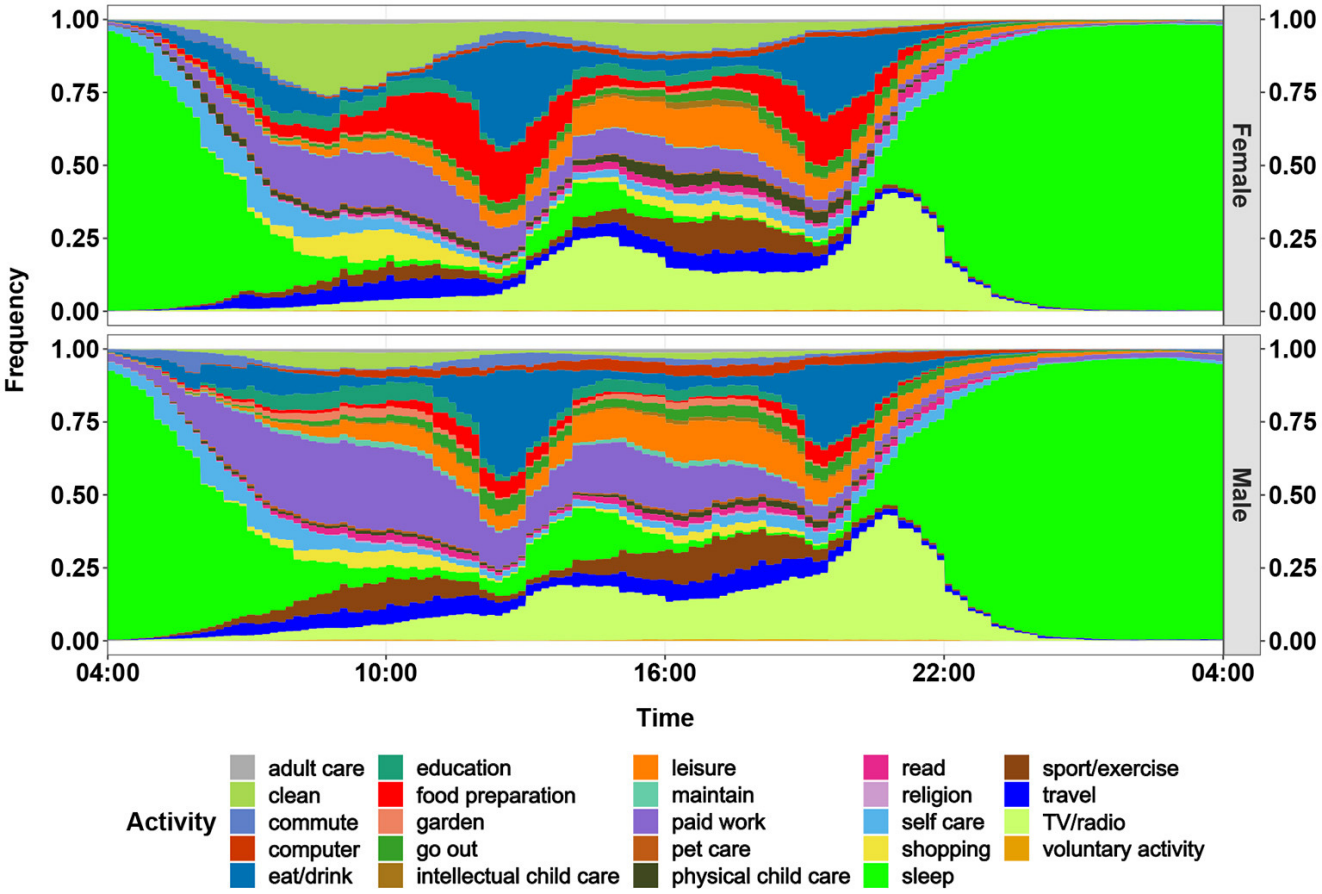
Daily activity profiles of females and males in Spain for 2010. The profiles (diaries) begin at 04:00 and end at 04:00 the next day. This figure displays the frequency of carrying out an activity for women and men for each minute of a day.

As an example, Figure 2 shows the time-activity patterns of Spanish males and females between 13 and 75 years old. Significant differences can be observed between the two groups, for instance the longer time women spent on cooking (marked red in the figure).

### 2.4. Life Course Trajectory Model

With the simulated concentration at different micro-environments and the time-activity pattern, it is able to assess the exposure during a defined year based on location, gender, age, and socioeconomic parameters of a person. However, for estimating the lifelong exposure of a person, the values of the socioeconomic status for each year of the person's life is needed, e.g., the educational or professional development.

Thus, a life course trajectory model has been developed within the frame of the EU project HEALS (50) and is applied here. The model was developed based on the EU-SILC (European Union Statistics on Income and Living Conditions) longitudinal data for employment status and education level (more details see Section 2.5.3), which documents the changes of the socioeconomic and employment status of individuals over their lifetime. The model was built with the TraMineR package of the R programming language (51). It enables the retrospective identification of the trajectory patterns of the education and professional status and the transitions between the states based on the status of a given year. To develop the life course trajectory model, the sequence analysis is used.

**Figure 3:**
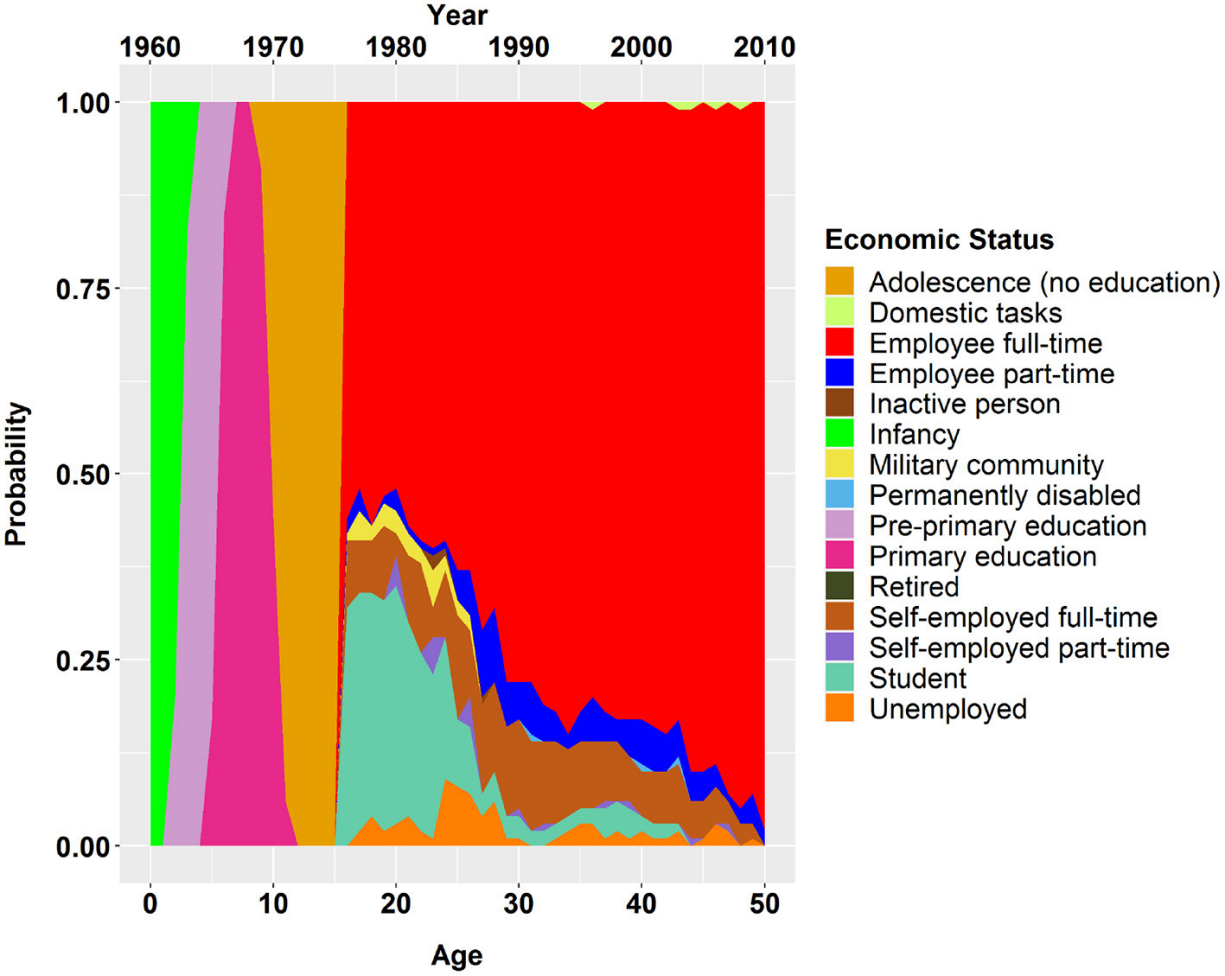
Life course trajectory of a German, male, full-time employee, age of 50 in 2010.

As an example for the result, Figure 3 shows the life course trajectory of a German male, full-time employee, aged 50 in 2010 as an example. Based on the information available for 2010 (50 years old, full-time employed), the model estimates a probability distribution of the educational or employment status for the previous life years of this person. Obviously, the more we look into the past, the more uncertainty occurs. But there are also time periods in youth where the status, e.g., primary education, are quite certain.

### 2.5. Health Impact Assessment

#### 2.5.1. Exposure Response Functions

As described in the Section 1, the state of the art methodology to assess health impacts associated with the exposure to air pollutants is to use CRFs, e.g., those published by the World Health Organization (WHO) for exposure to particulate matter, ozone and nitrogen dioxide (52). Using background incidence rates for the occurrence of the different health endpoints addressed in Europe, the relative risks from WHO can be transformed into concentration impact functions (CIFs), that explain how many additional cases of certain illnesses (e.g., lower respiratory symptoms) in absolute terms occur, or how many years of life are lost due to premature deaths, when 100,000 people in Europe are burdened with 1 additional μg/m^3^ of ambient background concentration for 1 year.

With describing a methodology for estimating exposures to pollutants over a life time for persons with certain socioeconomic status, we have provided an important prerequisite for developing improved impact functions. And for one of the drawbacks of using CRFs, the use of outdoor concentrations instead of exposures, we can—using certain assumptions—already show the effect of using the new exposure indicators instead to estimate associated health impacts.

We start with impact functions calculated in the EU HEIMTSA project (53). The CIFs that describe the emergence of diseases associated with fine particles in the parts of the respiratory system above the alveoli (e.g., chronic bronchitis, respiratory hospital admissions) use the PM2.5 concentrations as parameter, but the results also include impacts caused by coarser particles up to 10 μg/m^3^. Thus the results give a full picture of the damage caused by fine particles. Detailed information is given in Supplementary Table 1.

However, as explained above, the exposure is a better indicator for health impacts than the ambient concentration. Thus, the CIFs should be transferred into exposure impact functions (EIFs) to assess health impacts. The transformation of CIFs to EIFs is based on a method developed by (53) within the frame of the EU research project HEIMTSA. The assumption is made, that in the epidemiological studies used for estimating the CRFs, the differences of the part of the indoor concentration, that results from indoor sources are small, i.e., that the use of indoor sources and the ventilation habits in the different locations of a study are similar. This is plausible, as the studies usually compare health effects in locations in the same country or region and as smokers are excluded from the analysis. With this assumption, CIFs can be transformed into EIFs with Equation (12):


(12) $$\begin{array}{l} EIF=\frac{CIF}{r} \end{array}$$

where *EIF* is the exposure impact function, *CIF* is the concentration impact function and *r* is the ratio between the part of the average annual exposure that is caused by emissions of outdoor source, and the average annual ambient background concentration. To calculate the EIFs, the ratio *r* is calculated for each country separately according to the countries exposure and background concentration values.

For chronic diseases, especially chronic mortality, the available EIFs estimate annual health impacts based on the annual exposure. However, the hypothesis for the process of developing chronic health impacts is different, namely, that an exposure over many years first causes respiratory diseases, which lead to cardiovascular diseases, that finally cause premature deaths. Thus, an EIF using the past exposure over many years would be a better indicator for estimating health impacts than the annual exposure. However, as only CRFs and thus CIFs using annual exposure are available, we have to use the corresponding EIFs for the health impact calculation. Following Burnett et al. (54), we assume that the CRFs and thus the EIFs for PM2.5 have a no-effect threshold of 2.4 μg/m^3^. Burnett et al. (54) argue that this is the lowest concentration measured in epidemiological studies, so no information for lower concentration is available. Other experts, such as Papadogeorgou et al. (55) argue that a linear extrapolation with no threshold should be used in this situation for calculating the overall damage associated with PM2.5. Using no threshold would increase the DALYs and damage costs by less than 10%; this is included in the uncertainty analysis. The EIF used for estimating chronic mortality associated with PM2.5 is linear (from 2.4 μg/m^3^). For the relatively low exposure in EU countries linearity is an acceptable approximation (56).

#### 2.5.2. General Procedure

The health impact brought by a certain pollutant to a region is calculated following Equation (13):


(13) $$\begin{array}{l} HE_{i}=\overset{n}{\underset{r=1}{\sum }}\overset{m}{\underset{g=1}{\sum }}EXP_{g,r}\times EIF_{i}\times POP_{g,r} \end{array}$$

where *HE*_i_ is the health impact of endpoint *i, EXP*_g, r_ is the exposure of subgroup *g* in region *r, POP*_g, r_ is the number of people in subgroup *g* in region *r*.

To have a direct impression of the overall burden of a disease, the health impacts for different endpoints are aggregated into one measure, i.e., the disability adjusted life years (DALYs). The DALY consists of two parts, the years of life lost (YOLL) due to premature mortality and the years lost due to disability (YLD) (57). While 1 YOLL corresponds to 1 DALY, illnesses are transformed into DALYs using two parameters: the severity weight, which indicates how severe an illness is on a scale from zero (healthy) to one (dead), and the duration of the disease in fractions of a year (see Equation 14).


(14) $$\begin{array}{l} DALY=\overset{n}{\underset{i=1}{\sum }}HE_{i}\times DW_{i}\times DD_{i} \end{array}$$

where *DALY* is the total disability adjusted life years, *HE*_i_ is the health impact of endpoint *i, DW*_i_ is the severity weight of health endpoint *i* and *DD*_i_ is the duration of health endpoint *i*. A table with the parameters used can be found in Table 3.

**Table 3:** DALY weights and durations.

**Health impact**	**Pollutant**	**Weight**	**Duration**
Bronchodilator usage adults	PM2.5	0.22	0.00274
Bronchodilator usage children	PM2.5	0.22	0.00274
Cardiac hospital admissions	PM2.5	0.71	0.038
New cases of chronic bronchitis	PM2.5	0.099	10
Infant mortality	PM2.5	1	80
Lower respiratory symptoms adults	PM2.5	0.099	0.00274
Lower respiratory symptoms children	PM2.5	0.099	0.00274
Minor restricted activity days	PM2.5	0.07	0.00274
Restricted activity days	PM2.5	0.099	0.00274
Respiratory hospital admissions	PM2.5	0.64	0.038
Work loss days	PM2.5	0.099	0.00274
Years of life lost	PM2.5	1	1
Years of life lost	NO_2_	1	1
Prevalence of bronchitic symptoms in asthmatic children	NO_2_	0.22	0.00274

The different health endpoints can also be transferred into monetary values (see Equation 15). The monetary values per health impact are derived from contingent valuation studies, for example by asking about the willingness to pay to avoid a low risk of getting a certain disease.


(15) $$\begin{array}{l} DC=\overset{n}{\underset{i=1}{\sum }}HE_{i}\times MV_{i} \end{array}$$

where *DC* is the total damage costs, *HE*_i_ is health impact of endpoint *i* and *MV* is the monetary value of health endpoint *i*. A list with the monetary values per health endpoint used is given in Table 4.

**Table 4:** Monetary values for health impact endpoint.

**Endpoint**	**Mean (€)**	**SD (€)**
Bronchodilator usage adults	80	4
Bronchodilator usage children	80	4
Cardiac hospital admissions	2,990	847
New cases of chronic bronchitis	66,000	9,500
Infant mortality	4,485,731	168,000
Lower respiratory symptoms adults	57	0
Lower respiratory symptoms children	57	0
Minor restricted activity days	2,990	847
Restricted activity days	57	0
Respiratory hospital admissions	194	0
Work loss days	441	0
Years of life lost (PM2.5)	59,810	29,387
Years of life lost (NO_2_)	59,810	29,387
Prevalence of bronchitic symptoms in asthmatic children	80	44

#### 2.5.3. Population Data

As displayed by Equation (13), population data are necessary to assess the health impacts caused in a city or region. In this study, the population data are derived from three sources: EU-SILC data, UN data and LAU data.

- EU-SILC data
  EU-SILC is a dataset provided by EUROSTAT with the aim to collect comparable multidimensional microdata on income, poverty, social exclusion and living conditions for European countries. Two types of data are available, i.e., the cross-sectional and the longitudinal data. The cross-sectional data cover information on income, social exclusion, education and other living conditions over a given time period, while the longitudinal data concern the changes at the individual level over a 4-year time period (26). However, data are only available for years after 2004.

For population data before 2005, two additional data are employed:

- UN data
  The UN (United Nations) data are disaggregated by gender and 5-year age group for each year from 1950 to 2015 for each country. The data are spatially available at country level.
- LAU data
  EUROSTAT also provides population data spatially disaggregated at LAU2 level from 1961 to 2011 for every 10 years. LAU2 is the local administrative unit that consists of municipalities or equivalent units in European countries^5^.

For this study, the population data from the UN are utilized as basis and disaggregated spatially by the LAU data and socio-demographically by the EU-SILC data.

## 3. Results

### 3.1. The Overall Temporal Trend

#### 3.1.1. PM2.5

**Figure 4:**
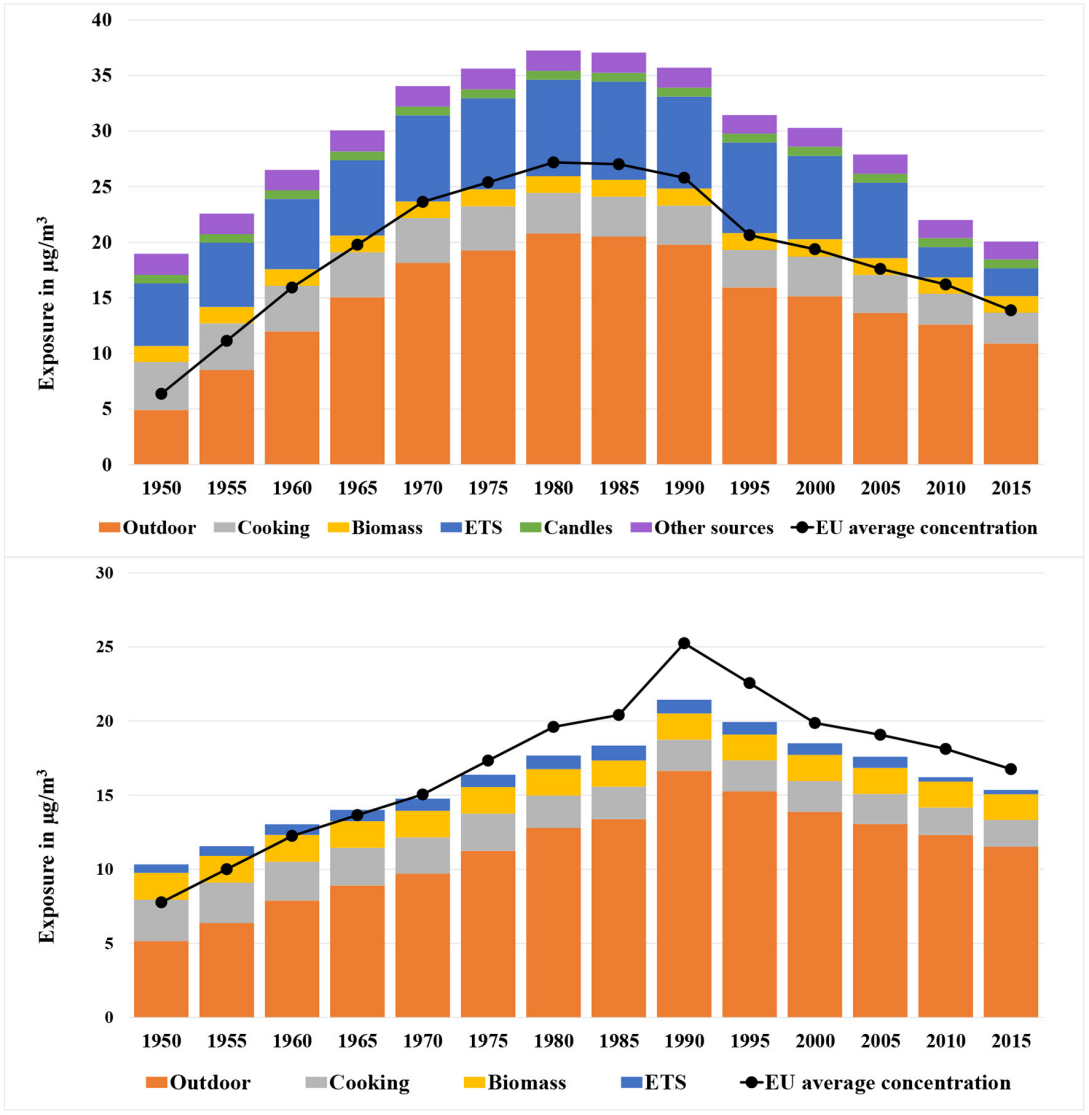
Population-weighted arithmetic mean PM2.5 (upper diagram) and NO_2_ (lower diagram) exposure by source (infiltration from outdoors, cooking, wood burning, smoking, candle/incense burning, and other sources) for European countries from 1950 to 2015. The black line indicates the average ambient background PM2.5/NO_2_ concentration in Europe near the home of people. The 95% CI of the exposure is mentioned in the text.

The mean exposures to PM2.5 and NO_2_ for an average European inhabitant were calculated for every 5 years from 1950 to 2015. Figure 4 (upper diagram) shows the population-weighted arithmetic mean PM2.5 exposure by source, including infiltration from outdoor environment, cooking, wood burning, smoking, candle/incense burning and other sources. The black line represents the average ambient PM2.5 concentrations for the European countries studied weighted with the population density. The total exposure to PM2.5 increased from 19.0 (95% CI: 3.3-55.7) μg/m^3^ in 1950 to a maximum of 37.2 (95% CI: 9.2-113.8) μg/m^3^ in 1980. After that, the exposure decreased to 20.1 (95% CI: 5.8-51.2) μg/m^3^ in 2015.

A high correlation can be seen between the overall exposure and the ambient background concentration. The average ambient background concentration in 1950 was relatively low at only 6.4 μg/m^3^. The value increased to 27.2 μg/m^3^ in the 1980s. Since the 1980s, a growing number of measures for reducing the emissions of fine particles and precursors (e.g., NO_x_, SO_2_) were implemented. The effect of these measures led to a continuous decline of the outdoor PM2.5 concentration to 13.9 μg/m^3^ in 2015.

Indoor sources contribute significantly to the exposure. The contribution was 74% in 1950, then decreased to 45% in 1980 because of the rapidly growing ambient concentration of PM2.5. The contribution made by indoor sources increased slowly until 2005 at 51%, when the exposure due to ETS began to drop and the contribution made by total indoor sources declined again to 46% in 2010.

The most important indoor source is passive smoking (ETS). In 1985, the exposure from ETS reached 8.8 μg/m^3^ and thus was responsible for over 20% of the total exposure. After 2005, the exposure was drastically reduced to only 2.5 μg/m^3^ in 2015. This was caused by a series of measures that were implemented in Europe to control the consumption of tobacco especially in public spaces, including regulation of tobacco products, advertising restrictions, creation of smoke-free environments, tax measures and activities against illicit trade^6^.

The second largest indoor source is cooking—more precisely frying and baking. An important process hereby is the evaporation of fat, that condensates in the air and thus builds fine particles. The contribution of cooking was slightly decreasing since firstly, people spent nowadays less time for cooking and secondly, the prevalence of kitchen hoods has been growing. Wood burning as another important indoor source has increased because of the growing importance of climate protection measures.

#### 3.1.2. NO_2_

Figure 4 (lower diagram) shows the mean NO_2_ exposure by source for Europeans from 1950 to 2015. The total exposure increased from 10.4 (95% CI: 0.9–36.8) μg/m^3^ in 1950 to a maximum of 21.4 (95% CI: 6.3–51.8) μg/m^3^ in 1990, and then decreased gradually afterwards to 15.5 (95% CI: 4.8–36.8) μg/m^3^ in 2015.

The most important outdoor source is road transport, followed by combustion of fuels. While larger combustion systems got more and more equipped with DENOX (SCR) filters, small wood stoves and in some regions coal stoves stayed as important NO_x_ emission sources.

In comparison to PM2.5, the contribution to exposure to NO_2_ caused by indoor sources is less dominant. In 1990, 22% of the exposure was caused by indoor sources. The ratio has increased only slightly to 25% in 2015. The main indoor sources were gas stoves operated in kitchens and biomass or coal stoves especially in some Eastern and Northern European countries.

### 3.2. Average Exposure at Country Level

Figure 4 shows the mean exposure averaged over all adult persons in Europe. However, the exposure of populations in different countries shows large differences. This effect will be analyzed in this section.

#### 3.2.1. PM2.5

- 2015
  Figure 5 (upper diagram) displays the arithmetic mean of PM2.5 exposure in 2015 for EU27+2 countries. The overall exposure as well as its source distribution varied considerably among different countries. Generally, the Eastern European (EE) countries have been burdened with the highest exposure to PM2.5. The reasons for this are:
  a) The EE countries have been suffered from quite high ambient background concentrations. For example, Bulgaria (18.6 μg/m^3^) and Poland (15.7 μg/m^3^) had the highest exposure caused by ambient concentrations, which is mainly caused by the use of coal.
  b) A larger percentage of people in EE countries were exposed to passive smoking (ETS) and wood burning indoors. For instance, in Lithuania and Poland, 30 and 24% of the people were exposed to ETS at home, while for Finland, this value was only 2%. Also, the exposure to wood burning was relatively high in EE countries. For example, 65% of the people in Estonia were exposed to wood burning at home.
  c) Last but not least, the dwelling sizes in EE countries were relatively small compared to other countries. According to the EU-SILC data, the average dwelling size in EE countries was 80 m^2^, which was 68% less than that of Northern European (NE) countries (134 m^2^). According to Equation (3), the small dwelling size reduces the dilution of indoor sources.
- 1980
  Figure 5 (lower diagram) displays the exposure to PM2.5 for the same European countries in 1980, when the average annual exposure reached a peak. At that time many Eastern and Southern European countries were not part of the EU and relied even more on coal as domestic energy carrier. Thus, the heaviest burdens were found in the EE countries such as Poland, Romania and Hungary. For Poland, the exposure to PM2.5 reached 66.2 μg/m^3^ and thus was the highest value in Europe.
  The most crucial contributor to the overall exposure was the penetration of pollutants in ambient air, especially for some EE countries, such as the Czech Republic (35.5 μg/m^3^), Poland (35.4 μg/m^3^), Hungary (32.1 μg/m^3^) and Slovakia (32.0 μg/m^3^).
  An important indoor source was smoking indoors. Countries like Greece (16.2 μg/m^3^), Cyprus (14.7 μg/m^3^) and Ireland (10.7 μg/m^3^) had a relatively large number of smokers.

**Figure 5:**
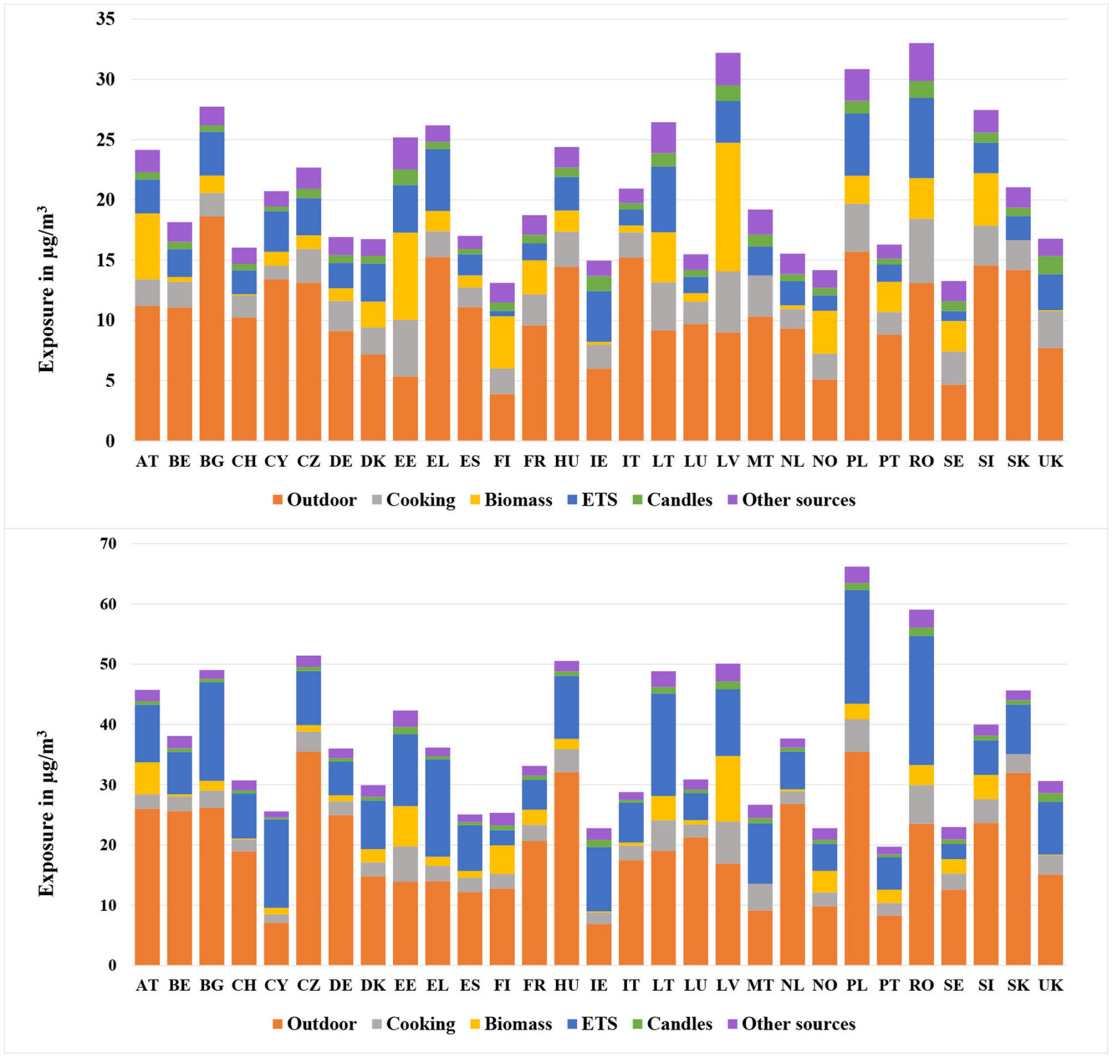
Population-weighted arithmetic mean PM2.5 exposure for different European countries in 2015 (upper) and 1980 (lower).

#### 3.2.2. NO_2_

- 2015
  Figure 6 (upper diagram) shows the NO_2_ exposure for European countries subdivided by source, including the infiltration from outdoors, cooking, wood burning and ETS. As mentioned in Section 3.1.2, infiltration from ambient environment was one of the most important sources for NO_2_ exposure. This conclusion applies particularly for some SE and NWE countries such as Italy (16.0 μg/m^3^), Germany (14.4 μg/m^3^), Belgium (14.1 μg/m^3^) and Luxembourg (14.0 μg/m^3^). In these countries the outdoor concentrations of NO_2_ in urban areas were extremely high. In contrast, the contribution made by outdoor air was not so remarkable in NE countries such as Finland (5.3 μg/m^3^) and Sweden (6.0 μg/m^3^), where the ambient concentrations were relatively low.
  Biomass was also significantly contributing to the overall NO_2_ exposure. For Latvia, the exposure due to biomass reached 11.9 μg/m^3^, which took 48% of its total exposure. The crucial contribution made by wood burning could also be found in countries like Estonia (7.7 μg/m^3^) and Austria (6.2 μg/m^3^).
  The contribution made by cooking varied considerably among different countries. EE countries, such as Romania (5.5 μg/m^3^), and Latvia (4.5 μg/m^3^) showed the largest exposure due to cooking among all the countries. In addition to the smaller dwelling size, the higher prevalence of gas and coal stoves was a reason for the relatively high exposure.
- 1990
  Figure 6 (lower diagram) shows the population-weighted arithmetic mean NO_2_ exposure by source for different European countries in 1990, when the overall exposure reached a maximum. Compared to other time periods, the contribution made by outdoor air was even higher. Especially for countries like Italy (22.2 μg/m^3^), United Kingdom (21.7 μg/m^3^), Germany (21.1 μg/m^3^), and Belgium (21.0 μg/m^3^), the infiltration took up almost 90% of the overall exposure. In comparison, the least substantial contribution made by infiltration was found in Ireland (6.8 μg/m^3^), Malta (7.4 μg/m^3^), and Cyprus (7.5 μg/m^3^). The most important indoor sources for NO_2_ exposure were cooking and wood burning, especially in Eastern European countries such as Latvia (17.6 μg/m^3^), Estonia (10.2 μg/m^3^), Romania (9.5 μg/m^3^), and Lithuania (7.4 μg/m^3^).

**Figure 6:**
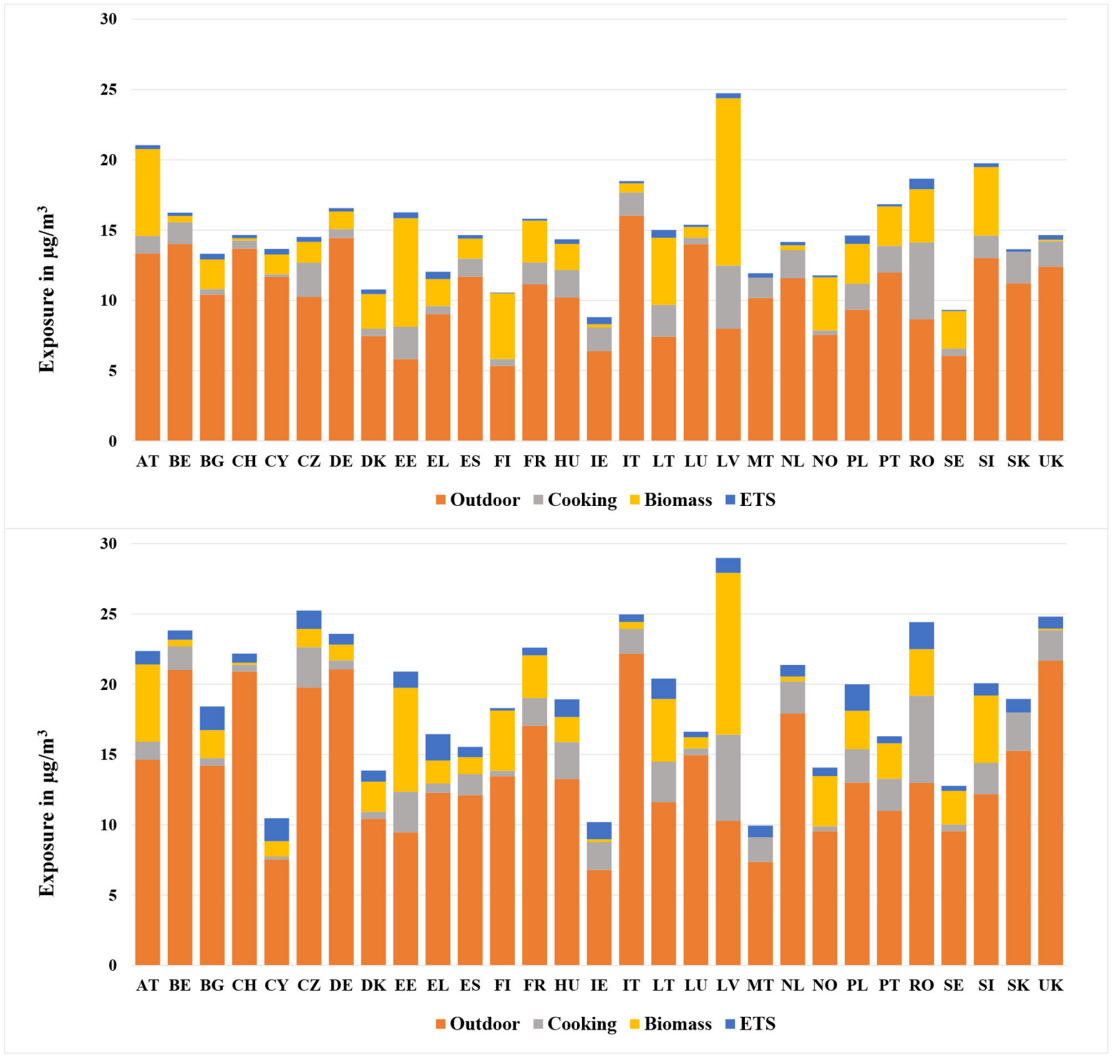
Population-weighted arithmetic mean NO_2_ exposure for different European countries in 2015 (upper) and 1990 (lower).

### 3.3. Exposure by Socioeconomic Status

The methodology allows to not only estimate the mean exposure of the population in a country, but also the exposure of population subgroups, that are characterized by certain features like gender, income and further parameters. Some of the detailed results are shown in this section.

#### 3.3.1. By Gender

**Figure 7:**
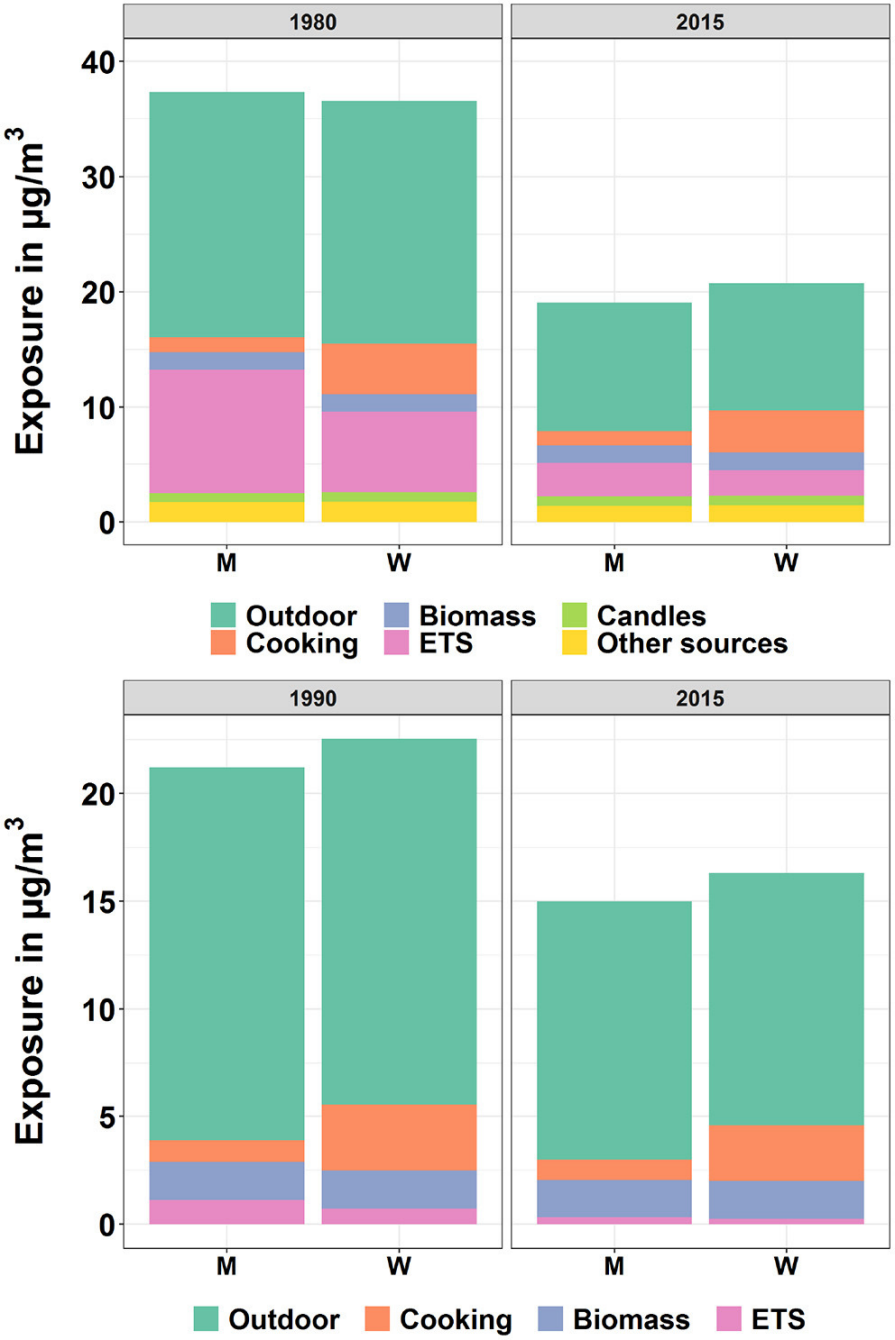
Population-weighted arithmetic mean PM2.5 (upper) and NO_2_ (lower) exposure by source and gender for European countries in 1980/1990 and 2015. “M” and “W” represent “Men” and “Women”, respectively.

- PM2.5
  Figure 7 (upper diagram) shows the population-weighted arithmetic mean PM2.5 exposure by source and gender for European countries in 1980 and 2015. In 1980, the overall exposure of men was higher than that of women. However, the opposite situation took place in 2015, when the exposure of men was lower than that of women.
  For PM2.5, the exposure due to cooking and ETS were the most important two indoor sources. According to the data from (58), the prevalence of male smokers was higher than that of females (35 to 25%), which obviously led to higher exposure from ETS experienced by men. In comparison, the exposure due to cooking experienced by women was much heavier than that of men. According to the MTUS data, the average time women spent on cooking was much higher than for men (see Figure 2), which resulted in higher exposure originating from cooking for women. The higher exposure due to ETS explained the precedence of men in 1980. After 2010, the exposure due to ETS was reduced significantly due to the Europe-wide introduction of smoking bans. Thus, cooking became more influential and the exposure for women became larger than that of men after 2010.
- NO_2_
  Figure 7 (lower diagram) shows the temporal development of population-weighted arithmetic mean NO_2_ exposure by source for both genders in European countries in 1990 and 2015. The NO_2_ experienced by women was higher than by men for both time periods. The main reason was the much higher exposure due to cooking experienced by women. As mentioned above, women spent much longer time on cooking than men, and this resulted in the heavier exposure experienced by women. However, a decrease of the difference between man and women can be noticed.

#### 3.3.2. By Income

**Figure 8:**
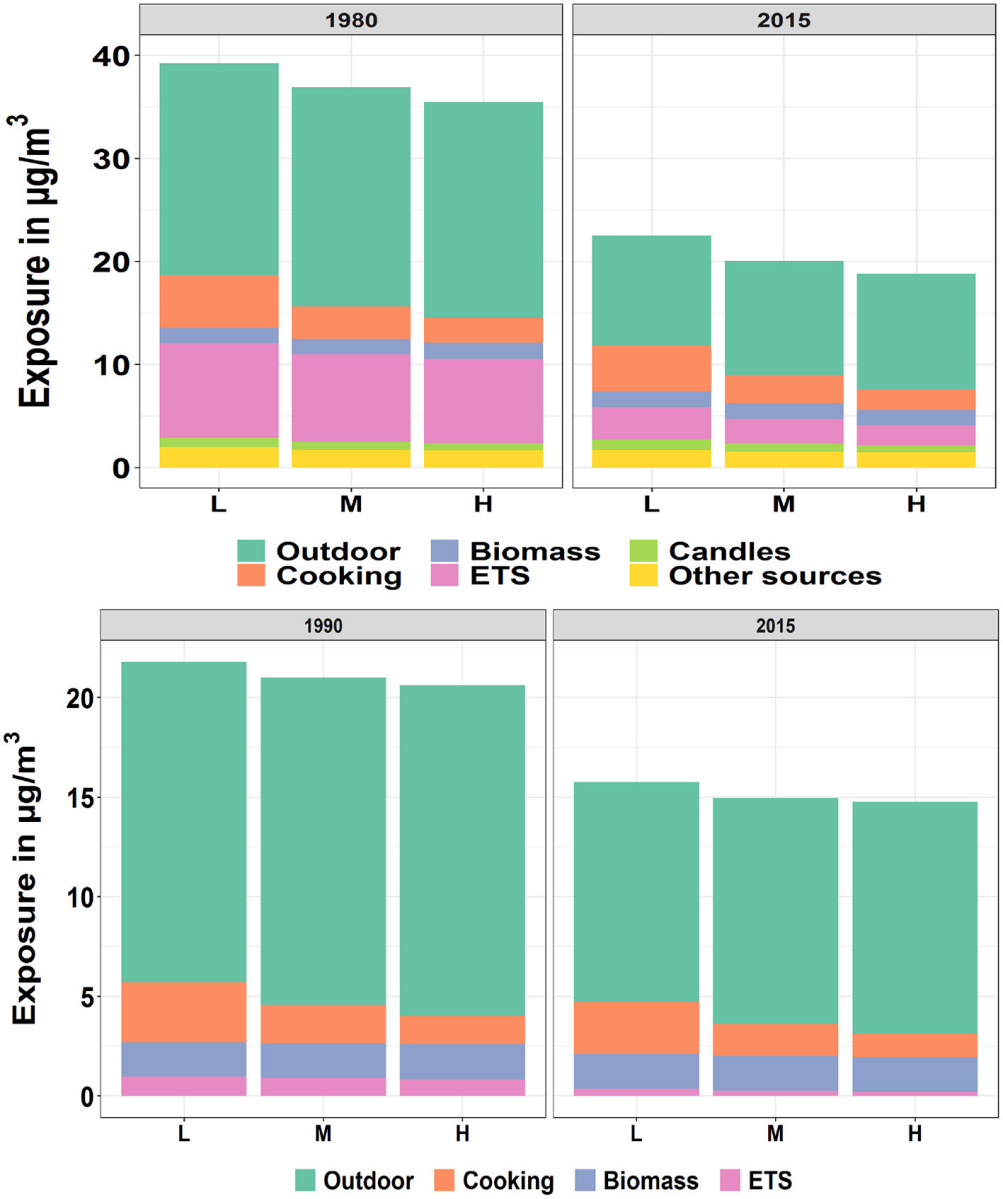
Population-weighted arithmetic mean PM2.5 (upper) and NO_2_ (lower) exposure by source and income level for European countries in 1980/1990 and 2015. The “L”, “M” and “H” represent the “Low”, “Median”, and “High” level of income, respectively.

- PM2.5
  Figure 8 (upper diagram) shows the average PM2.5 exposure for three levels of household income. As displayed by the figure, the subgroup with the lowest income level was exposed to the highest exposure among the three subgroups. The difference of exposure due to cooking among the three groups was noticeable. There are two reasons responsible for this outcome. First of all, the people from the lowest income group tended to live in smaller dwellings, which led to a smaller dilution of pollutants indoors. Secondly, according to the MTUS data, the cooking time was negatively correlated to the income level, as for instance higher income groups ate more in restaurants. For other indoor sources, the higher exposure by low income group could also be observed.
- NO_2_
  Figure 8 (lower diagram) shows the mean NO_2_ exposure by source and income level for Europeans in 1990 and 2015. Similar to the temporal development of PM2.5 for the three income levels, the exposure to NO_2_ was also found to be highest by the group with the lowest income. The most important cause for the difference among the three groups was cooking.

Similar results are calculated for the exposure differentiated according to education level and employment status, as these parameters are closely correlated to income.

### 3.4. Health Impact Assessment, DALYs, and Damage Costs

**Figure 9:**
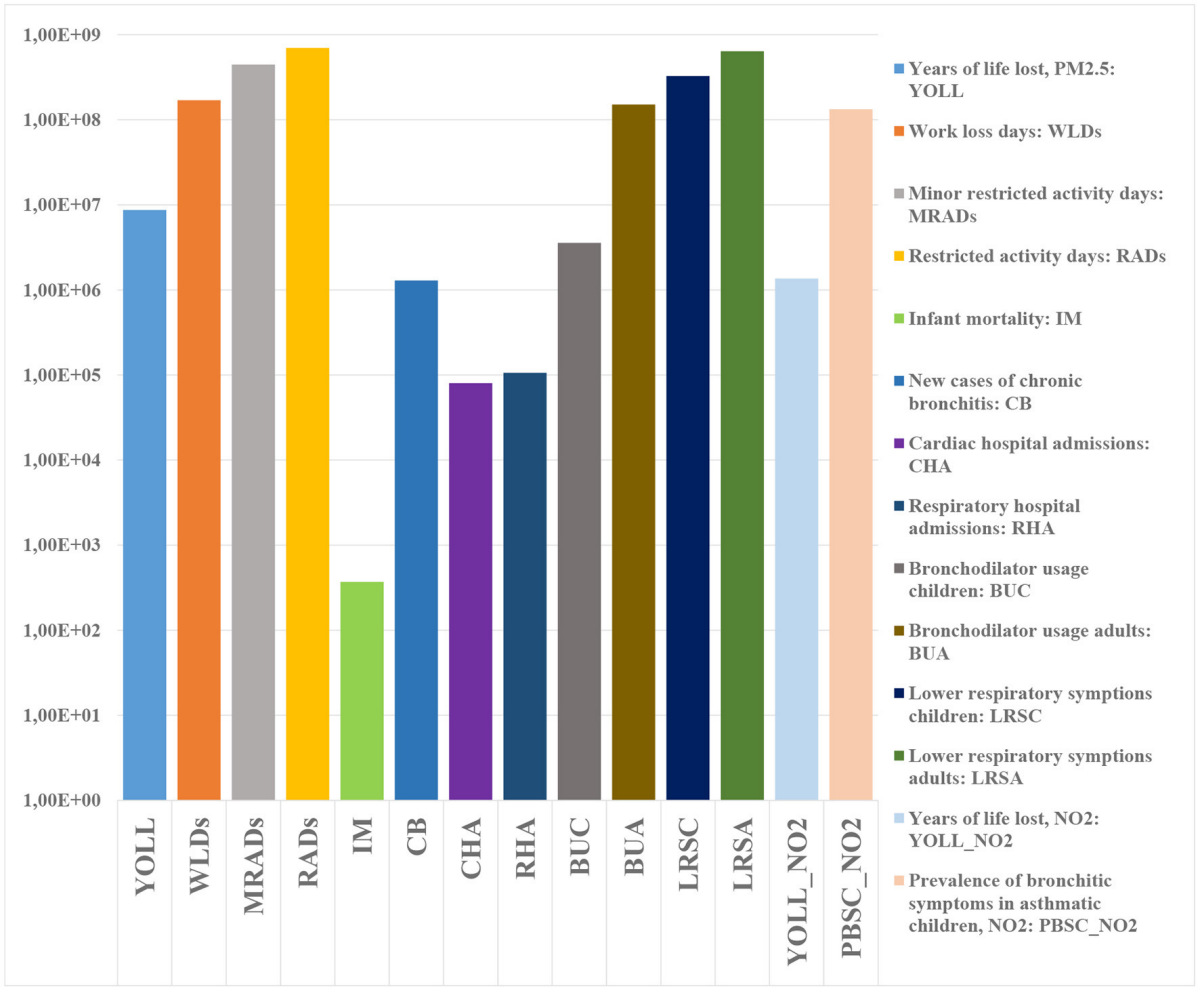
Health impacts for the European population in 2015.

Figure 9 shows the result of the health impact assessment with the help of EIFs. As reported by WHO (2013a), the long-term impacts due to NO_2_ partly overlap with those caused by fine particles. Due to this reason, an overlap factor of 33% is applied for NO_2_ in this study, which means, that the calculated chronic health impacts, DALYs and damage costs caused to NO_2_ are reduced by one third.

**Figure 10:**
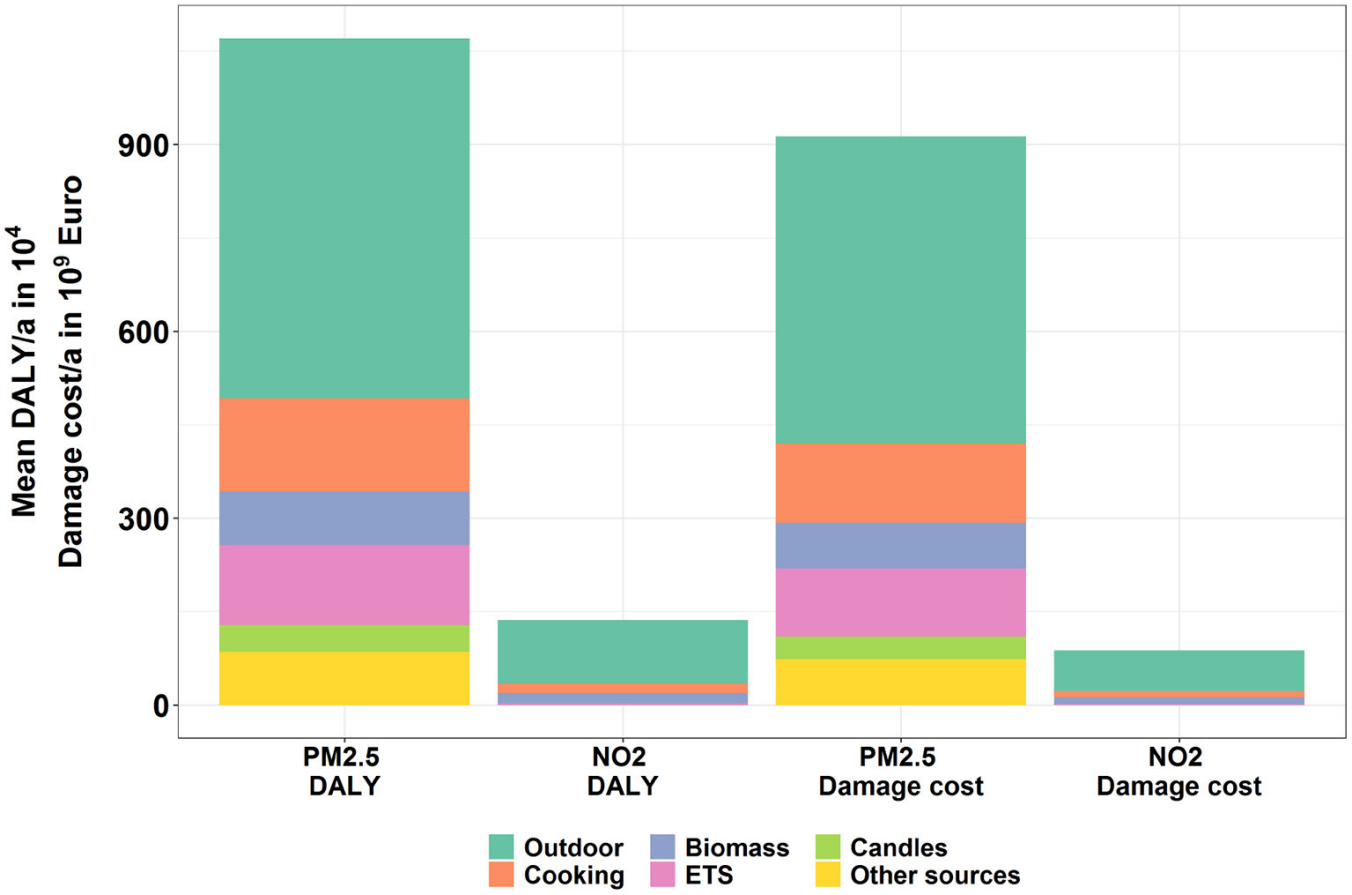
Overall DALYs and damage costs caused by PM2.5 and NO_2_ emissions of the EU countries in 2015.

In a next step, health impacts are transferred into DALYs and damage costs. Figure 10 displays the total DALYs and damage costs caused by PM2.5 and NO_2_ for Europeans in 2015. The total DALYs and damage costs amounted 1.22 × 10^7^ (95% CI: 3.61 × 10^6^-2.95 × 10^7^) DALYs and 1.01 × 10^12^ (95% CI: 3.46 × 10^11^-2.37 × 10^12^) *e*, respectively, within which 88.1% of the DALYs and 90.8% of the damage costs were stemming from PM2.5. Among all the health endpoints, YOLL due to PM2.5 was the most important contributor, which accounted for 71.8 and 52.0% of the total DALYs and damage costs, respectively.

### 3.5. Lifelong Exposure

In Figure 4, the average exposures of the European population to both pollutants for every fifth year from 1950 to 2015 are presented. In this section, the authors follow a person over the years of his or her lifetime. Thus not only the concentration of pollutants in micro-environments, but also the changes of the educational and socioeconomic status of a person are taken into account to estimate the exposure of a certain person over his/her lifetime.

**Figure 11:**
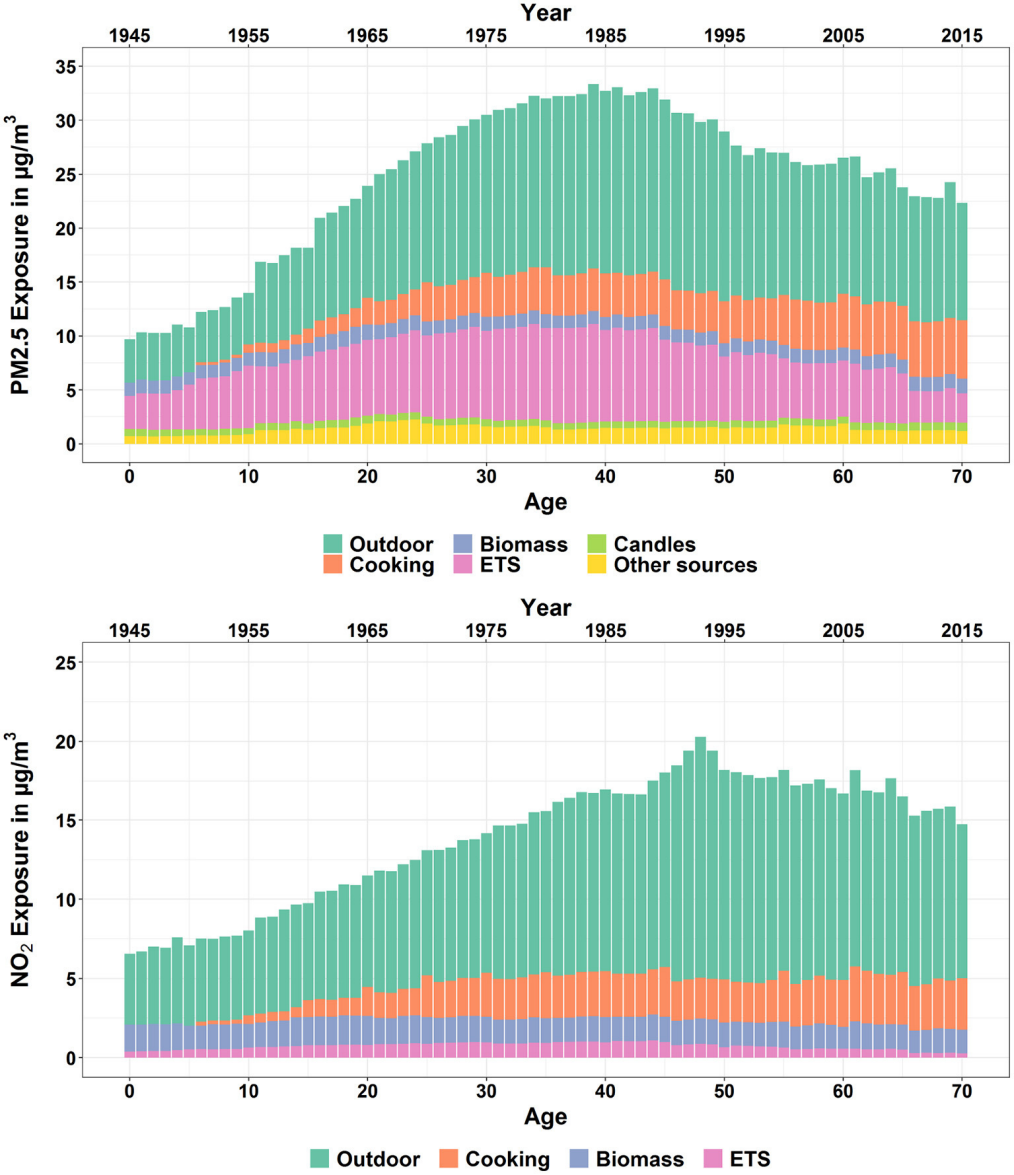
Temporal course of the average lifelong exposure to PM2.5 (upper) and NO_2_ (lower) for an 70-year-old European differentiated by source, including infiltration from outdoors, biomass, candles, cooking, ETS (passive smoking), and other sources.

Figure 11 show the temporal courses of the lifelong exposure to both pollutants differentiated according to the source for an average European person who was 70 years old in 2015. For this person, the average lifelong exposure to PM2.5 and NO_2_ was 24.86 (95% CI: 2.75–83.17) and 14.22 (95% CI: 1.35–44.80) μg/m^3^. The contribution of outdoor sources to this lifelong exposure was 47% for PM2.5 and 67% for NO_2_. The contribution from cooking was increasing for both pollutants which was caused by the fact that the cooking time spent increases with age. The annual average exposure to PM2.5 and NO_2_ caused a reduction of life expectancy (in years of life lost) of 3.68 × 10^-2^ (95% CI: 4.99 × 10^-3^−1.22 × 10^-1^) and 5.01 × 10^-3^ (95% CI: 0.00–3.19 × 10^-2^) for each year of exposure of the analyzed person from age 30 onwards. Transformed into days of life lost, this results in a reduction of life expectancy of 14.65 (95% CI: 1.82–52.29) days per year of exposure.

## 4. Conclusion and Outlook

Even though large efforts have been made to improve air quality, Europe still faces burdens associated with air pollutants, especially by PM2.5 and NO_2_. Clearly, the most important burdens in the context of air pollution are health impacts. The environmental policy instruments used by the European Commission to reduce the damage are on one hand the Air Quality Directives, that regulates the concentrations of pollutants at certain monitoring stations, that are sited outdoors at busy streets or in the background of urban and rural areas. On the other hand, the emissions of sources emitting to outdoor air are regulated with various directives.

Obviously, these instruments are not optimal with regard to reducing the health impacts. Humans are affected by air pollutants by inhaling pollutants into their lung. Thus the concentration of pollutants at places where people breath are the parameters that are directly correlated with health impacts. In other words, the exposure of people to pollutants rather than the concentrations at certain outdoor places with monitoring station would be an appropriate indicator for health impact assessment, especially as the people in Europe spend most of the time indoors.

Of course there is a certain correlation between the outdoor and indoor concentration. But using the outdoor concentration as an indicator for health effects leads to the ignorance of two other important parameters: the contribution of indoor sources to the exposure, and the influence of air exchange rates between outdoor and indoor air and filters on the exposure.

Thus, the authors describe and apply here a methodology that is capable of estimating the lifelong exposure of persons and population subgroups with certain features like gender, age, location of workplace and home, education and socioeconomic status.

A probabilistic framework, which contains a life course trajectory model, a time-activity model, a mass-balance model and atmospheric models has been established to simulate the long-term exposure. The outcome of the framework, i.e., the lifelong exposure of persons with certain features like gender, age, location of home and work and socioeconomic status, is then combined with EIFs, aggregation factors and monetary values to assess the health impacts and damage costs.

For the exposure assessment, several indoor sources, including infiltration from outdoors, biomass, candles, cooking, ETS and other sources have been considered. The annual average exposure to PM2.5 for European countries showed a trend of continuous increase from the 1950s at 19.0 (95% CI: 3.3–55.7) μg/m^3^ to a peak in the 1980s at 37.2 (95% CI: 9.2–113.8) μg/m^3^. After the 1980s the exposure turned to decrease until 2015 at 20.1 (95% CI: 5.8–51.2) μg/m^3^. Similarly, the exposure to NO_2_ started to increase from the 1950s at 10.4 (95% CI: 0.9–36.8) μg/m^3^ to the highest point at 21.4 (95% CI: 6.3–51.8) μg/m^3^, and then began to decrease gradually until 2015 at 15.5 (95% CI: 4.8–36.8) μg/m^3^. The drop of the exposures after the peak years can be explained by the implementation of a series of policies in Europe to reduce the outdoor emissions of the air pollutants since the 1970s. Additionally, for PM2.5, the introduction of the policies for tobacco control has alleviated the exposure due to ETS significantly. For NO_2_ the role played by ETS was much less prominent, while cooking and wood burning indoors were the most crucial indoor sources.

As a main result, it turned out, that emissions of indoor sources have caused on average about 50% of the total exposure to PM2.5 and 31% to NO_2_, respectively. This proves that a very considerable part of health impacts associated with air pollution is neglected in the current air pollution policy.

A large variance can be observed in the exposures in different countries. For PM2.5, the heaviest burdens are mainly experienced in Eastern European countries. In these countries, lignite has been a main energy carrier for a long time and indoor smoking and use of coal stoves were much more predominant than in Western European countries. In addition, the average dwelling size in Eastern European countries were comparably small, which slowed down the dilution of the indoor pollutants.

The influence of the socio-demographic factors, including gender, income level, employment status, as well as education level were discussed in this study. With respect to gender, men experienced higher exposure due to environmental tobacco smoke (ETS) as a result of the higher prevalence of smokers and tobacco consumption. In contrast, women were burdened with heavier exposure due to cooking because of their longer stay in the kitchen. Regarding income, the population with the lowest income level experienced the highest level of exposure. This outcome was mainly due to the smaller dwelling size and longer cooking time of the lower-income group. This finding also holds for the influence of education level and employment status.

The estimation of the lifelong exposure described here is an important prerequisite for developing better health impact functions. To demonstrate the use of the results, health impacts associated with air pollution have been calculated with EIFs, that are derived by transforming the commonly used CIFs.

With the EIFs health impacts due to the exposures to PM2.5 and NO_2_ have been also assessed and then integrated as DALYs and damage costs. In 2015, the exposure to PM2.5 and NO_2_ in European countries resulted in 1.22 × 10^7^ (95% CI: 3.61 × 10^6^−2.95 × 10^7^) DALYs and 1.01 × 10^12^ (95% CI: 3.46 × 10^11^−2.37 × 10^12^) € damage costs, respectively. 88.1% of the DALYs and 90.8% of the damage costs were due to the exposure to PM2.5.

The authors have also simulated the lifelong exposure to both pollutants for individuals with certain features. As an example, for an average European aged 70 in 2015, the average exposure over his or her lifetime to PM2.5 and NO_2_ was 24.86 (95% CI: 2.75–83.17) and 14.22 (95% CI: 1.35–44.80) μg/m^3^, respectively. The exposure to both pollutants led to YOLLs (years of life lost) of 3.68 × 10^-2^ (95% CI: 4.99 × 10^-3^−1.22 × 10^-1^) and 5.01 × 10^-3^ (95% CI: 0.00–3.19 × 10^-2^) per year of exposure, i.e., an average loss of life expectancy of 0.44 (95% CI: 0.06–1.46) and 0.06 (95% CI: 0.00–0.38) month, respectively, per life year exposed from the age of 30 onwards. Summing this up over 40 years (from age 30 to 70) results in a reduction of life expectancy of 19.3 (95% CI: 2.39–68.76) months. This result is larger than earlier estimates, as it includes the health impacts caused by emissions of indoor sources.

The results highlight the importance of not only reducing emissions from outdoor sources, but also those from indoor sources. Some examples for measures, whose usefulness can be derived from the results shown above: especially the high emissions from frying in the kitchen demand for the use of efficient cooker bonnets. Other important sources are candles and incense sticks, whose use should be more restricted. Open chimneys and older wood stoves should be replaced with the state of the art stoves. Smoking indoors should be reduced as much as possible. Rooms should be regularly cleaned with vacuum cleaners equipped with an HEPA filter. If buildings are retrofitted with new tight windows, the building should be equipped with a decentral mechanical ventilation with heat recovery.

The uncertainties are quite high. Many assumptions had to be made, especially when using the indoor model. More data on emissions rates, air exchange rates and ventilation behavior would be helpful. A differentiation of the transport micro-environment into separate modes would also be useful. More indoor emission sources should be explicitly modeled, e.g., toasters, laser printers, hair blowers, cleaning agents and personal care products. A major still unknown issue is the process of the development of chronic diseases. The available concentration response relationships correlate annual average concentrations with chronic diseases, especially chronic mortality. However, the authors assume that chronic mortality develops over many years, starting with respiratory symptoms that get chronic and then cause cardiovascular symptoms that finally lead to premature deaths. Thus epidemiological studies that correlate lifelong exposure with chronic mortality would be needed. Further studies should analyze the effects of multi-pollutant exposure and of the influence of number, size and content of species of particles.

## Data Availability Statement

The original contributions presented in the study are included in the article/Supplementary Materials, further inquiries can be directed to the corresponding author/s.

## Author Contributions

NL and CS designed the model and the computational framework. NL analyzed the data, carried out the implementation, and performed the calculations. NL and RF wrote the manuscript using input from all authors. RF conceived the study and was in charge of overall direction and planning. Both authors contributed to the article and approved the submitted version.

## Funding

This work was supported by the European Union within the research projects HEALS (FP7, grant agreement No. 603946) and ICARUS (H2020, grant agreement No. 690105).

## Conflict of Interest

The authors declare that the research was conducted in the absence of any commercial or financial relationships that could be construed as a potential conflict of interest.

## Publisher's Note

All claims expressed in this article are solely those of the authors and do not necessarily represent those of their affiliated organizations, or those of the publisher, the editors and the reviewers. Any product that may be evaluated in this article, or claim that may be made by its manufacturer, is not guaranteed or endorsed by the publisher.

## Footnotes

^1^The European Union in the period between 2007 and 2013, including the UK and before Croatia joined, when it had 27 countries.

^2^ https://emep.int/mscw/mscw_moddata.html

^3^ https://www.eea.europa.eu/data-and-maps/data/airbase-the-european-air-quality-database-6

^4^ https://www.openstreetmap.org

^5^ https://ec.europa.eu/eurostat/web/nuts/local-administrative-units

^6^ https://ec.europa.eu/health/tobacco/overview_en
