# Supplementary material for: Exposure of Individuals in Europe to Air Pollution and Related Health Effects
Source: Front Public Health. 2022 May 25;10:871144. doi: 10.3389/fpubh.2022.871144 (PMC9174982; doi:10.3389/fpubh.2022.871144)
Supplement: Supplementary file 1 [file Table_1.pdf]

# Supplementary Material

## 1 TABLES

Table S1: Overview of the impact functions.

| Health impact | Pollutant | Age group | Concentration Impact function |
|---|---|---|---|
| Bronchodilator usage adults (BUA) | PM2.5 (>2.4 $\mu g/m^3$) | 20+ | 14,600 (± 14,150) additional days of bronchodilator usage per 1 $\mu g/m^3$ increase in PM2.5 per 100,000 adults aged 20 and older with well-established asthma per year. |
| Bronchodilator usage children (BUC) | PM2.5 (>2.4 $\mu g/m^3$) | 5-14 | 2,200 (± 6,025) additional days of bronchodilator usage per 1 $\mu g/m^3$ increase in PM2.5 per 100,000 children aged 5-14 years meeting the PEACE study criteria per year. |
| Cardiac hospital admissions (CHA) | PM2.5 (>2.4 $\mu g/m^3$) | All | 0.65 (± 0.16) additional emergency cardiac hospital admissions per 1 $\mu g/m^3$ increase in PM2.5 per 100,000 people (all ages) per year. |
| New cases of chronic bronchitis (CB) | PM2.5 (>2.4 $\mu g/m^3$) | 18+ | 14 (± 5.95) new cases of chronic bronchitis per 1 $\mu g/m^3$ increase in PM2.5 per 100,000 at-risk adults aged 18 and older per year. |
| Infant mortality (IM) | PM2.5 (>2.4 $\mu g/m^3$) | 1 month to 1 year | 0.87 (± 0.29) additional infant deaths per 1 $\mu g/m^3$ increase in PM2.5 per 100,000 live births per year. |
| Lower respiratory symptoms adults (LRSA) | PM2.5 (>2.4 $\mu g/m^3$) | 18+ | 20,800 (± 8,750) additional lower respiratory symptom days per 1 $\mu g/m^3$ increase in PM2.5 per 100,000 adults aged 18 and older with chronic respiratory symptoms per year. |
| Lower respiratory symptoms children (LRSC) | PM2.5 (>2.4 $\mu g/m^3$) | 5-14 | 29,000 (± 7,400) additional lower respiratory symptom days per 1 $\mu g/m^3$ increase in PM2.5 per 100,000 children aged 5-14 per year. |
| Respiratory hospital admissions (RHA) | PM2.5 (>2.4 $\mu g/m^3$) | All | 0.86 (± 0.0625) additional emergency respiratory hospital admissions per 1 $\mu g/m^3$ increase in PM2.5 per 100,000 people (all ages) per year. |

*Continued on next page*

Table S1 – *Continued from previous page*

| Health impact | Pollutant | Age group | Impact function |
|---|---|---|---|
| Minor restricted activity days (MRAD) | PM2.5 (>2.4 μg/$m^3$) | 18-64 | 5,770 (± 545) additional MRAD per 1 μg/$m^3$ increase in PM2.5 per 100,000 adults aged 18-64 (general population) per year. |
| Restricted activity days (RAD) | PM2.5 (>2.4 μg/$m^3$) | 18-64 | 9,020 (± 552.5) additional RAD per 1 μg/$m^3$ increase in PM2.5 per 100,000 adults aged 18-64 (general population) per year. |
| Work loss days (WLD) | PM2.5 (>2.4 μg/$m^3$) | 18-64 | 2,070 (± 155) additional work loss days per 1 μg/$m^3$ increase in PM2.5 per 100,000 people aged 15-64 in the general population per year. |
| Years of life lost (YOLL_PM2.5) | PM2.5 (>2.4 μg/$m^3$) | 30+ | 101.4 (± 7.5) additional YOLL per 1 μg/$m^3$ increase in PM2.5 per 100,000 people aged 30 and older in the general population per year. |
| Years of life lost (YOLL_$NO_2$) | $NO_2$ (>20 μg/$m^3$) | 30+ | 93.0 (± 6.9) additional YOLL per 1 μg/$m^3$ increase in $NO_2$ per 100,000 people aged 30 and older in the general population per year. |
| Prevalence of bronchitic symptoms in asthmatic children (PBSC) | $NO_2$ | 5-14 | 111,427 (± 2,128) additional bronchitic symptom days per 1 μg/$m^3$ increase in $NO_2$ per 100,000 children aged 5-14 per year. |
